# Surgical and Functional Outcomes of Artery Only Versus Artery and Vein Clamping in Patients Undergoing Partial Nephrectomy: A Systematic Review and Meta-Analysis

**DOI:** 10.5152/tud.2022.22009

**Published:** 2022-05-01

**Authors:** Abdalla Ali Deb, Ayman Agag, Naufal Naushad, Hosam Serag

**Affiliations:** 1 Department of Urology, The James Cook University Hospital, Middlesbrough, UK; 2 Department of Urology, Frimley Park Hospital, Camberely, UK; 3 Department of Urology, Wythenshawe Hospital, Manchester, UK; 4 Department of Urology, University Hospitals Birmingham, Birmingham, UK

**Keywords:** Kidney, neoplasms, nephrectomy

## Abstract

Clamping of renal vessels during partial nephrectomy is usually performed to improve the visualization of tumor margins. However, clamping of renal vessels has been associated with detrimental effects on renal function after surgery. This study aimed to compare artery only versus artery and vein clamping as regards the surgical and functional outcomes in patients undergoing partial nephrectomy. The literature was searched for English published studies from January 1, 2000 to August 7, 2021. The search included MEDLINE/PubMed, Cochrane Library, Scopus, Web of Science, Google Scholar, and ProQuest, using the terms {“partial nephrectomy”} OR {“nephron-sparing surgery”} AND {“renal artery and vein clamping} AND {“renal artery only clamping}. Nine studies were included. Meta-analysis showed the artery only clamping group had a significantly less percentage of change in glomerular filtration rate at last follow-up (standardized mean difference: −0.42 [95% CI: −0.70, −0.13], *P* = .004) as well as a rate of postoperative complications (odds ratio: 0.64 [95% CI: 0.41, 0.98], *P* = .04). However, no significant difference was observed regarding the development of chronic kidney disease. There was no significant difference regarding the warm ischemia time, blood loss, or positive surgical margin. Artery only clamping has a comparable safety to artery and vein clamping and may produce a renoprotective effect. Due to limitations of the included studies, the conduction of large-size randomized clinical trials with a long duration of follow-up is required before recommending the replacement of artery and vein clamping with artery only clamping during partial nephrectomy.

**Main Points**

- Artery only (AO) clamping and artery and vein (AV) clamping have comparable safety regarding the estimated blood loss, transfusion rate, and positive surgical margin.
- AO clamping may produce a renoprotective effect compared to AV clamping.
- Before replacing AV clamping with AO clamping, further clinical trials are required with a longer duration of follow-up to avoid the limitations of the available studies.

## Introduction

Current guidelines recommend partial nephrectomy for the management of small renal tumors that are confined to the kidneys.^1,2^ Partial nephrectomy can be performed by different approaches, including the traditional open surgical, the laparoscopic, and the robotic-assisted approaches. In addition, several surgical techniques exist.^3^ Clamping of the renal vessels is a step that is performed in many techniques of partial nephrectomy. There is a debate whether it is more beneficial to clamp both the renal artery and vein (AV) clamping or to clamp the artery only (AO) clamping. Some studies even suggest that segmental or selective clamping of the renal artery could improve the patients’ outcomes. The proposed benefit of AO clamping, compared to AV clamping, is reducing the renal ischemic changes by allowing retrograde venous perfusion. Meanwhile, the AV clamping blocks venous backflow during excision of the tumor, thereby improving the visualization of the margins of the tumor margins and enhancing renal reconstruction.^4-6^

The superiority of AO clamping to AV clamping during partial nephrectomy was reported mainly by studies conducted on animal models,^5-8^ which reported better tolerance to renal ischemia. Nevertheless, most studies on human subjects did not report more beneficial effects of AO clamping compared to AV clamping.^4,5,9,10^ These controversial results lead to a lack of consensus on the best technique of clamping during partial nephrectomy.

The present study was conducted to fill this gap considering the benefits of one technique of clamping over the other. This systematic review and meta-analysis were carried out to compare AO and AV clamping as regards the surgical and functional outcomes of patients undergoing partial nephrectomy.

## Methods

### Methodology

The protocol of this systematic review and meta-analysis was registered at the International Prospective Register of Systematic Reviews (PROSPERO) (registration number: CRD42021277651) on September 16, 2021.

The conduction of this systematic review and meta-analysis followed the principles of the *Cochrane Handbook for Systematic Reviews of Interventions*, version 6, and was reported according to the Preferred Reporting Items for Systematic Reviews and Meta-Analyses (PRISMA) guidelines.^11^

### The Research Question

Is AO clamping superior to AV clamping in adult patients undergoing partial nephrectomy as regards the surgical and functional outcomes?

### Research Aim and Objectives

This systematic review and meta-analysis aims to compare AO versus AV clamping in adult patients undergoing partial nephrectomy, with the following objectives: (a) to assess the clinical characteristics of patients undergoing AO or AV clamping during partial nephrectomy; (b) to compare the surgical outcome; and (c) to compare the functional outcome.

### Inclusion Criteria for Studies

**Types of Studies:** Observational (cohort or case-control) studies and clinical trials were included in this systematic review and meta-analysis. The search was restricted to studies published in English during the period from January 1, 2000 to August 7, 2021.

**Participants:** Included studies were conducted on adult patients who had partial nephrectomy.

**Interventions:** Eligible studies included a direct comparison between patient groups undergoing AV clamping and AO clamping.

**Exclusion Criteria:** The following types of publications were excluded: conference abstracts/posters, duplicate reports, case report, review articles, editorials, commentaries, and clinical guidelines. In addition, studies conducted on animals or patients less than 18 years old as well as studies not including a direct comparison of both types of interventions were excluded.

### Search Strategy

**Electronic searches:** A search of the electronic databases of MEDLINE/PubMed, Cochrane Library, Scopus, Web of Science, Google Scholar, and ProQuest was conducted. The search was limited to studies published in English during the period from January 1, 2000 to August 7, 2021. The search was conducted during the period from July 21, 2021 to August 7, 2021.

The used search terms were {“partial nephrectomy”} OR {“nephron-sparing surgery”} AND {“renal artery and vein clamping} AND {“renal artery only clamping}.

**Other Resources:** A search was conducted for potentially relevant studies that were identified from the reference lists of studies retrieved from electronic search.

### Selection of Studies

One reviewer carried out the research and then screened the titles and abstracts of retrieved studies. The full text of the studies with potentially relevant abstracts was obtained and screened for eligibility by the same reviewer using the aforementioned inclusion and exclusion criteria. The second reviewer checked the results of the search, the screening process of titles and abstracts, as well as the review of the full text of potentially eligible studies. Any disagreement was settled by the third reviewer.

### Data Extraction

One reviewer extracted relevant data from the included studies using a standardized datasheet. The extracted data included: (a) the study characteristics (the country, study design, duration of the study, the number of patients, and the duration of follow-up after surgery); (b) patients’ characteristics (age, sex, body mass index (BMI), comorbidities, size and stage of the tumor, and neoadjuvant/adjuvant therapy, preoperative estimated glomerular filtration rate [eGFR]); (c) the surgical details: approach (open, laparoscopic, or robotic-assisted), types of clamping, duration, estimated blood loss; and (d) postoperative complications, duration of follow-up, postoperative complications, and postoperative eGFR. The second reviewer checked the collected data to ensure consistency and clarity. Disagreements were resolved by consulting the third reviewer. No blinding was used for the journal titles, authors, or institutions.

### Measured Outcomes

**Primary Outcome:** It comprises the surgical and functional outcomes of AV and AO clamping. The surgical outcome included warm ischemia time, transfusion rate, estimated blood loss during surgery, and operative time. The functional outcome included the levels of creatinine and glomerular filtration rate after surgery and the rate of newly developed chronic kidney disease (CKD) after the surgery.

**Secondary Outcomes:** Secondary outcomes comprised the clinical characteristics of patients, including their age, sex, comorbidities, body mass index, and the type of surgical approach (open, laparoscopic, or robotic-assisted partial nephrectomy).

### Assessment of the Risk of Bias in Included Studies

The risk of bias was assessed for included studies by one reviewer using the National Institute for Health and Care Excellence (NICE) checklist for cohort studies and randomized clinical trials.^12^

### Data Synthesis

Review Manager (RevMan Version 5.4. The Cochrane Collaboration, 2020) was used for performing the analysis, computing standardized metrics, and producing forest plots. For each comparison, the number of studies showing a positive direction of effect and the number of studies with statistically significant effects were reported.

Categorical dichotomous outcomes (e.g., complications, CKD) were expressed as odds ratio (OR) and 95% CI were calculated. An OR > 1 indicated a higher risk in the AO clamp group, while an OR < 1 indicated a higher risk in the AV clamp group. Continuous numerical variables (e.g., warm ischemia time) were summarized for each study as the standardized mean difference (SMD) as a measure of effect size by subtracting the mean for the AV clamping group from the mean for the AO clamping group then dividing the result by the pooled standard deviation. A positive value of SMD indicated an increase of the outcome in the AO group relative to the AV group, while a negative SMD value indicated a decrease in the AO group. The effect size was classified according to the rule of thumb by Cohen^13^ (for ORs: large ≥ 4.3; medium ≥ 2.5, small ≥ 1.5, negligible < 1.5; for SMDs: large ≥ 0.8; medium ≥ 0.5, small ≥ 0.2, negligible < 0.2).

The extracted data were tested for heterogeneity using the Cochrane chi-square heterogeneity test and *I* ^2^ index. Significant heterogeneity across the studies was determined at a Cochrane chi-square test with a *P* value of <.1 and an *I* ^2^ index ≥50%. If testing for heterogeneity yielded nonsignificant results, pooling of the extracted data was performed using the fixed-effect model.^14^ If significant heterogeneity was detected, the random-effects model was used. For interpreting the comparisons between the groups, a *P* value of <.05 was considered significant.

## Results

### Results of Literature Search and Study Selection

**Figure 1.**
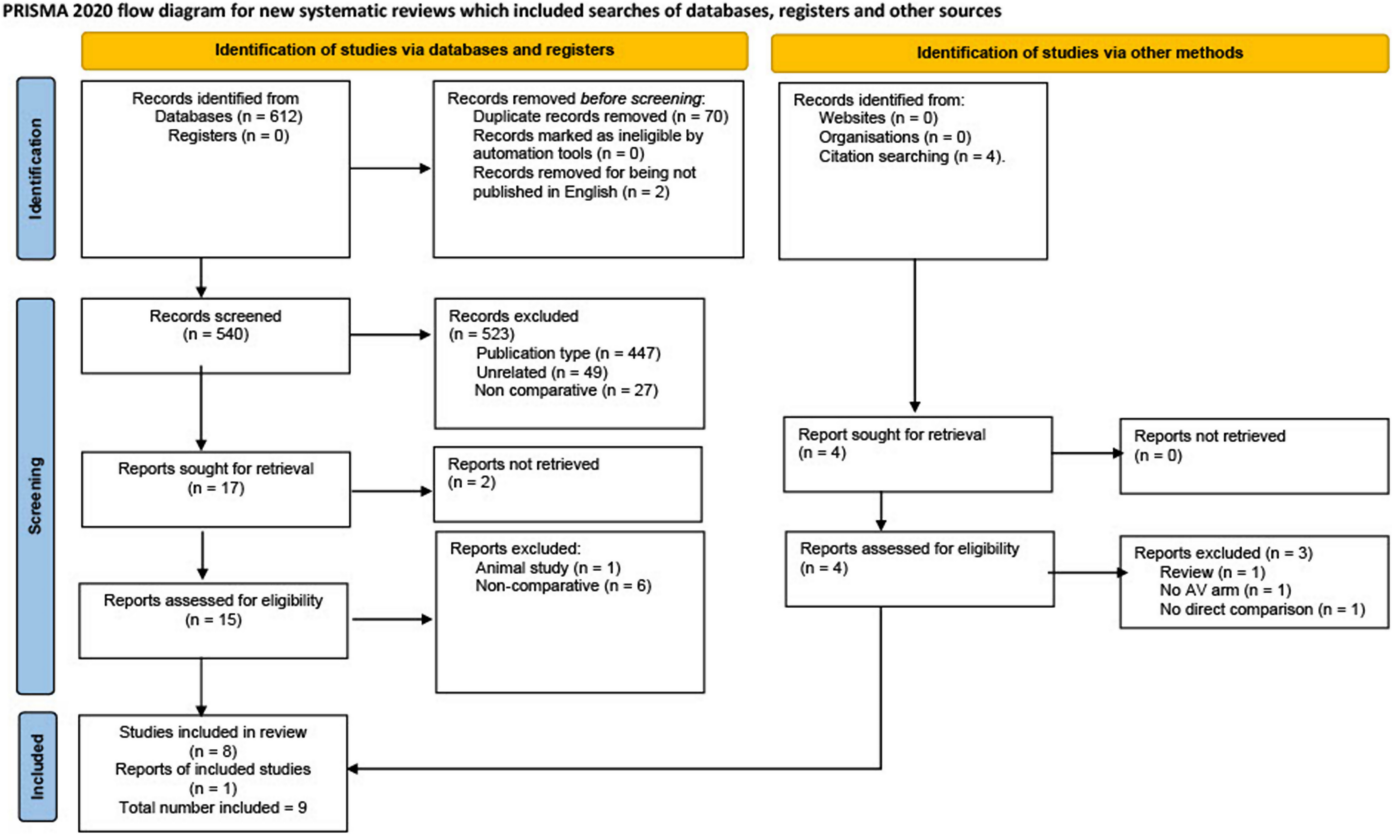
The PRISMA flow chart for the results of literature search and study selection. PRISMA, Preferred Reporting Items for Systematic Reviews and Meta-Analyses.

The literature search yielded 610 articles. The process of screening titles, abstracts, and full-text studies is illustrated in the PRISMA 2020 flowchart (Figure 1).

A total of 69 duplicate results were removed and 2 studies were found to be published in a language other than English. A total of 540 records were then screened as regards their titles and abstracts, with the result of excluding 523 records due to their publication type (n = 447), nonrelevance (n = 49), or the lack of comparisons between the interventions of interest (n = 27).

The full text of 17 records was sought for retrieval: 2 articles were not available. Screening of the available 15 records ended by the exclusion of 7 studies (1 animal study and 6 noncomparative studies that included 1 arm only of the studied interventions). Eight studies were then found to be eligible for inclusion.^4,9,10,15-19^ Screening of the reference lists of retrieved articles led to the identification of 4 relevant studies, out of whom 3 were excluded (1 review article, 1 study not including an AV clamping group, and 1 not including direct comparison of AO vs AV clamping), and 1 article was eligible.^20^ The overall number of included studies in this systematic review was 9.

### Basic Characteristics and Assessment of the Risk of Bias of the Included Studies

**The Basic Characteristics of the Included Studies:** Tables 1 and 2 demonstrate the characteristics of the included studies. All studies were conducted in a single center, except the study by Blum et al^9^. Out of the included 9 studies, 2 were randomized clinical trials.^18,19^ The remaining 7 studies were observational cohort studies: 5 were retrospective,^9,10,15-17^ and 2 were prospective.^4,20^

The countries where the studies were conducted included the United States,^9,17,20^ Canada,^10^ Japan,^4^ Turkey,^15,16^ Korea,^18^ and Switzerland.^19^ The surgical approach of partial nephrectomy was open surgery in 2 studies,^18,20^ laparoscopic partial nephrectomy (LPN) in 3 studies,^4,10,17^ robot-assisted partial nephrectomy (RAPN) in 1 study,^9^ and a mixed sample of LPN and RAPN in 3 studies.^15,16,19^

**Summary of the Included Studies:** Gong et al^17^ conducted a single-center, retrospective, cohort study in the United States on patients undergoing LPN from October 2002 to May 2006. The exclusion criteria included previous or subsequent extirpative renal surgeries. In addition, they excluded patients with the open conversion from the analysis of postoperative renal function. The number of patients was 25 in the AO clamping group and 53 in the AV clamping group. Assessment of the renal function included serum creatinine and creatinine clearance, which were measured before surgery, immediately after surgery, then on the first postoperative day (POD1), and at the last follow-up visit. They defined the development of CKD as the serum creatinine level above 1.4 mg/dl or a creatinine clearance below 60 ml/min. The eGFR was not assessed. The mean follow-up after surgery was significantly longer in the AO group than the AV group (21.9 vs 10.1 months, respectively, *P* < .001). They found that creatinine and creatinine clearance significantly changed in the AV group compared to the baseline levels, but not in the AO group. The two groups were comparable regarding the rate of blood transfusion, positive surgical margin (PSM), and warm ischemia time.

Imbeault et al^10^ carried out a single-center, retrospective, cohort study in Canada on surgically fit patients with localized enhancing renal masses who underwent LPN during the period from March 2003 to December 2008. No exclusion criteria were reported. Their cohorts consisted of 103 patients with AO clamping and 102 patients with AV clamping. Assessment of renal function included measurement of serum creatinine and eGFR preoperatively, at POD1, 3 months, and at last follow-up visit. In addition, they assessed split renal function using mercaptoacetyl triglycine (MAG)-Lasix scintigraphy in 62 patients. However, they did not report on the rate of development of CKD after surgery. The duration of follow-up was significantly longer in the AO group (median 44 vs 15 months, respectively, *P* < .001). They reported that the mean warm ischemia time was significantly longer in the AO group (30.4-8.2 vs 23.3 minutes—10.0, *P* < .001) and had a significantly higher change in postoperative eGFR (13.7 ml/min vs 10.2 ml/min, *P* = .047). The two groups showed a comparable average loss of differential renal function, estimated blood loss (EBL), operative time, and rate of postoperative complications. They conducted a multivariate analysis and found that the clamping technique was not significantly contributing to the reduction in renal function.

The cohort study by Liu et al^20^ was prospective in design and was conducted on patients who had a potentially malignant renal mass on computed tomography/magnetic resonance imaging and underwent complex open PN as they were unfit for LPN or RAPN because of the size or site of the tumor. Arterial only clamping was done in 12 patients while AV clamping was performed in 25 patients. Renal function was assessed using serum creatinine and eGFR, which were measured preoperatively, postoperatively, and at last follow-up. In addition, they assessed renal oxygenation during clamping using digital light processing-hyperspectral imaging. They found no significant difference between the two groups as regards their renal oxygenation profiles during PN. The median follow-up period was nonsignificantly longer in the AO group (6.8 vs 2.7 months, *P* = .68). There was no significant difference between the two groups regarding the postoperative change in renal function.

Funahashi et al^4^ included in their prospective cohort study patients with nonhilar exophytic renal tumors undergoing LPN from August 2005 to January 2013. They excluded patients with endophytic and hilar tumors. The method of clamping was AO in 32 patients and AV in 26 patients. Renal function was assessed using serum creatinine, eGFR, as well as MAG3 scintigraphy (preoperatively, 1 week, and 6 months postoperatively). The duration of follow-up was 6 months for both groups, but they did not report losses to follow-up. They found that postoperative renal function was comparable between the two groups and that reduction in renal function correlated with warm ischemia time. More reduction in function was found in the AV group patients who had a warm ischemia time of 25 minutes or longer compared to similar patients in the AO group.

Blum et al^9^ conducted a multicenter, retrospective cohort study on patients undergoing RAPN from 2008 to 2016 who had a solitary T1 renal mass, a functional contralateral kidney, baseline eGFR ≥ 30, and available follow-up data for 3 to 18 months post-surgery. They excluded cases in which surgeons performing AV clamping in less than 10% of surgeries were involved. The methods of clamping were AO in 70 patients and AV in 163 patients. Assessment of renal function included percent change in eGFR and acute kidney injury (AKI) at discharge and development of CKD at 9 months post-RAPN. They defined progression to CKD as “an increase from CKD stage 1 or 2 to CKD stage ≥3 or an increase from CKD stage 3 to CKD stage ≥4 at a median follow-up of 9 months.” The median follow-up duration was nearly similar in AO and AV groups (9.3 and 8.7 months, *P* = .413). They also conducted a propensity score-matched analysis. They found that patients with AO clamping had a significantly longer warm ischemia time, but no significant difference was found regarding EBL, transfusion rate, postoperative complications, postoperative renal function, or progression to CKD.

Artykov et al^16^ evaluated in their retrospective study patients with solitary, unilateral, and cT1 renal masses who underwent LPN or RAPN during the period from 2008 to 2019. They excluded patients with off-clamp PN, conversion to open surgery, compelled radical nephrectomy, missing clamping data, and those lost to follow-up. Patients undergoing AO clamping were 41 while those undergoing AV clamping were 27. They assessed serum creatinine and eGFR and recorded the rate of CKD after surgery. They diagnosed progression to CKD when postoperative eGFR was below 60 ml/min/1.73 m^2^. The reported median follow-up period for all subjects was 13.5 months, but the individual follow-up for each group was not reported. They found that AO clamping was superior to AV clamping in terms of preserving renal function and rates of progression to CKD, but operative time was significantly longer in the AO group.

Song and Jang^18^ carried out a randomized clinical trial on patients with T1 renal tumor undergoing open PN from 2015 to 2018 in a single center. Their sample size included 43 patients in the AO group and 45 in the AV group. For assessment of renal function, serum creatinine was measured before surgery, then on POD1, 7, 15, and then on first and third-month post-surgery. In addition, differential renal function was assessed 3 months after surgery, but they did not report on the rate of progression to CKD. The duration of follow-up was 3 months for both groups, but losses to follow-up were not stated. They found that the reduction in renal function was less in the AO group until POD7, but after that, the two groups had similar measurements of renal function. They concluded that AO clamping is not superior to AV clamping considering the long-term renal outcome.

Another randomized clinical trial was conducted by Würnschimmelet al^19^ in a single center on patients with cT1-T2 renal masses. They excluded patients with a Charlson Comorbidity Index above 10, CKD stages 4-5, previous renal surgeries or concomitant oncological diseases, immune diseases, cT3+ or cN1 renal cancer. One arm of the study included 61 patients undergoing RAPN with AO clamping, while the other arm included 54 patients allocated for LPN with AV clamping. Renal function was assessed using MAG3 renal scintigraphy preoperatively and at 6 months follow-up. Patients were followed up for 6 months (no loss to follow-up in the AV group but 5 losses to follow-up in the AO group). They found that RAPN with AO clamping had a longer mean operative time than LPN with AV clamping. Otherwise, no significant differences were found regarding warm ischemia time, the rate of transfusion, PSM, complications, and reduction in renal function.

The most recent included study was a retrospective, single-center, cohort study by Akpinar et al^15^ on patients who underwent open or minimally invasive PN. Their exclusion criteria were anatomical malformation of kidney, preoperative CKD, conversion to RN, undergoing simultaneous intraabdominal surgeries, bilateral renal masses, solitary kidney, follow-up <2 years, as well as zero or segmental ischemia. The AO group comprised 154 patients while the AV group consisted of 192 patients. Renal function was assessed by the estimation of eGFR at the 6th, 12th, and 24th months post-surgery. Progression to CKD was defined as an increase from baseline CKD stage 1 or 2 to ≥3. Patients were followed up for 2 years. They reported the lack of significant difference in renal functions between the AO and AV clamping groups regardless of the surgical approach. Multivariate analyses showed that AKI was significantly associated with renal score and preoperative eGFR, while progression to CKD at 2 years was significantly associated with older age and preoperative eGFR. They concluded that AO clamping is not more beneficial than AV clamping for preserving postoperative renal function.

**Figure 2.**
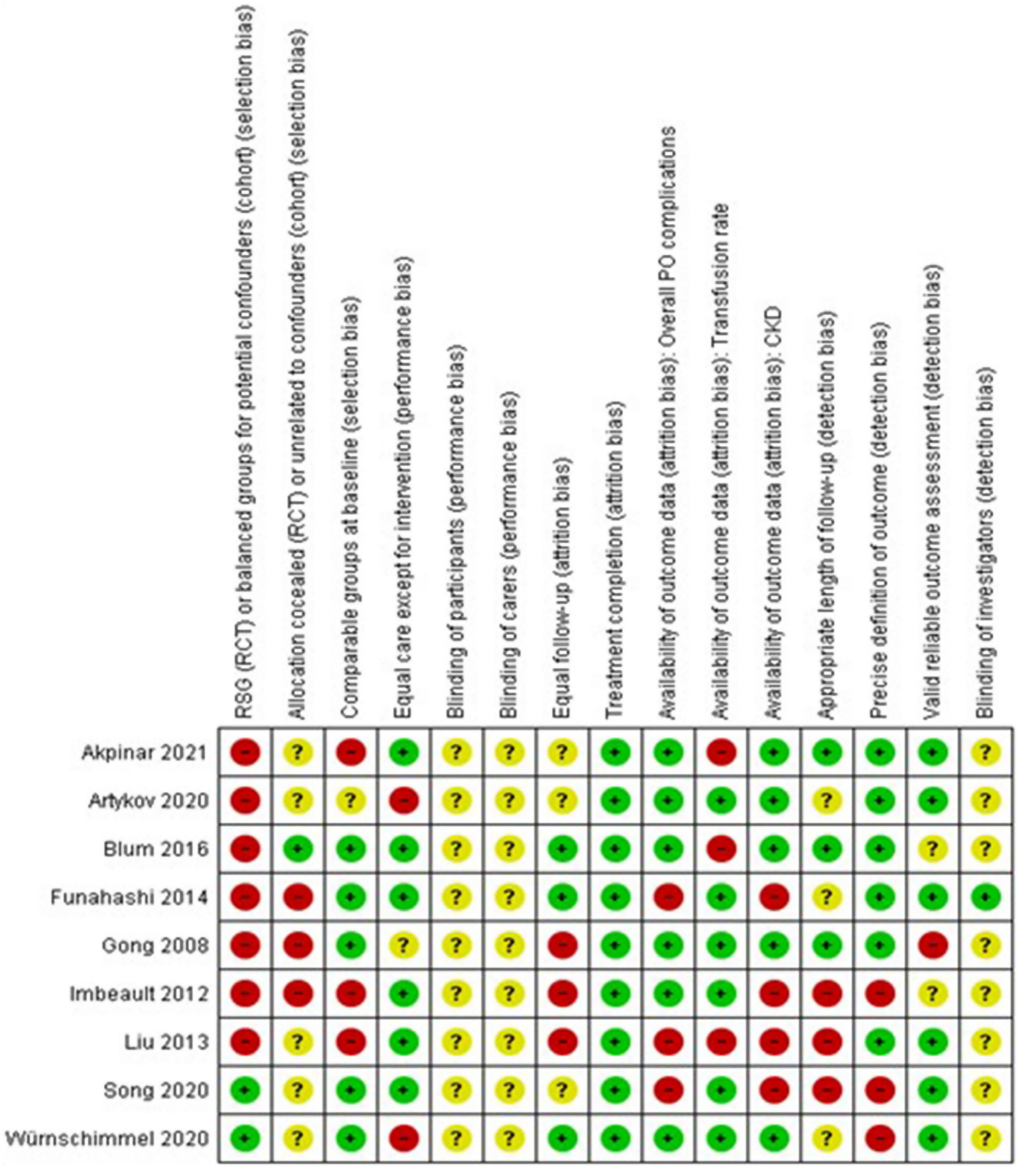
Risk of bias summary as assessed for each study. RCT, randomized controlled trial; RSG, random sequence generation.

**Figure 3.**
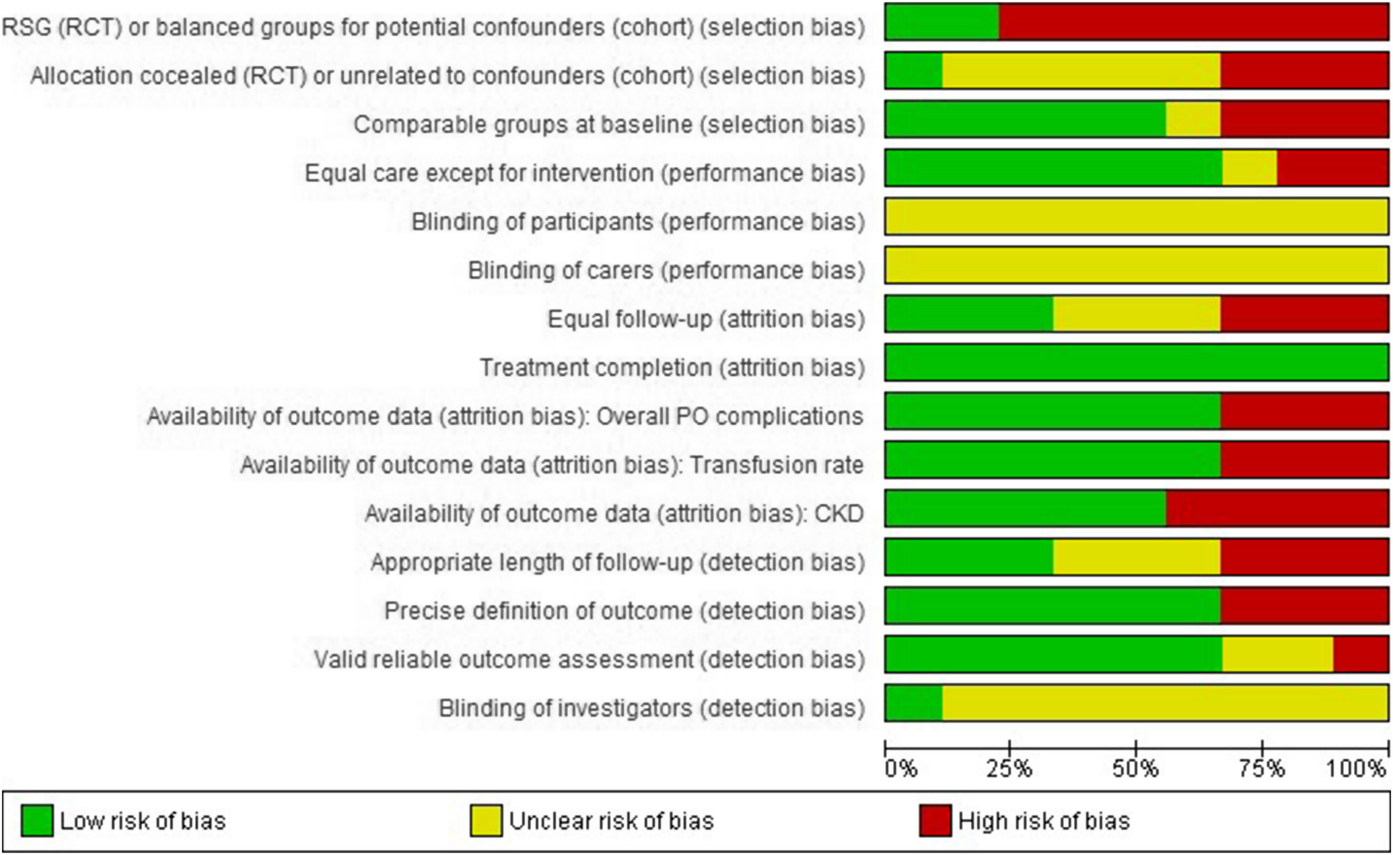
Risk of bias summary of each item presented as percentages across all included studies. RCT, randomized controlled trial; RSG, random sequence generation.

**The Assessment of the Risk of Bias in the Included Studies:** Assessment of the risk of bias was performed using the NICE checklist for cohort studies and randomized clinical trials (Figures 2 and 3), which comprised four main domains: selection, performance, attrition, and detection biases.

As regards the selection bias, the two clinical trials^18,19^ showed a low risk of bias regarding the random sequencing generation; however, the allocation concealment showed uncertain risk as neither study mentioned the process of allocation. The potential of confounders affecting the choice of intervention was uncertain or even high (particularly in those studies with significant differences in baseline characteristics between the two groups) in most observational studies, except for the study by Blum et al.^9^ which dealt with this possibility by conducting a propensity score-matched analysis. The two groups were not comparable before the intervention in 3 studies. Imbeault et al^10^ reported that the technical skills of the surgeon and the size of tumors were higher in the AV group, the latter factor may impact the warm ischemia time and the amount of removed renal tissue, thereby affecting postoperative renal function. A significantly larger mean tumor size was reported in the AO group by Liu et al^20^ and nonsignificantly larger in the AV group by Artykov et al^16^ Akpinar et al^15^ reported that the AV group had a significantly higher mean age, tumor size, and renal score. Overall, a high risk of selection bias was found.

Regarding the performance bias, most studies reported equal care for the two groups except Artykov et al^16^ who mentioned a higher percentage of LPN in the AV group; Gong et al^17^ who did not mention the operative details and care; and Würnschimmel et al^19^ who used a different surgical approach for each group. None of the studies reported blinding of the patients or carers while blinding of the investigators was reported in one study only.^4^

When the attrition bias was assessed, we found that the duration of follow-up of the AO group was longer than that of the AV group in 3 studies,^10,17,20^ while three studies did not state the follow-up duration for each group.^15,16,18^ A high risk of bias was particularly found regarding the reporting of the development or progression of CKD.

As for the detection bias, the length of follow-up was relatively short in some studies,^10,18,20^ some cases that may later progress to CKD may have been missed. The outcome was properly assessed in most studies, except for the Gong et al^17^ study, which depended on serum creatinine and creatinine clearance without estimation of eGFR or measurement of MAG scintigraphy.

## Results of Narrative Synthesis and Meta-Analysis

Studies were grouped for narrative synthesis and meta-analysis according to the surgical approach of PN: open^18,20^ or minimally invasive (LPN and/or RAPN).^4,9,10,16,17,19^ One study^15^ included patients who underwent various surgical approaches, and its results are presented separately.

**Figure 4.**
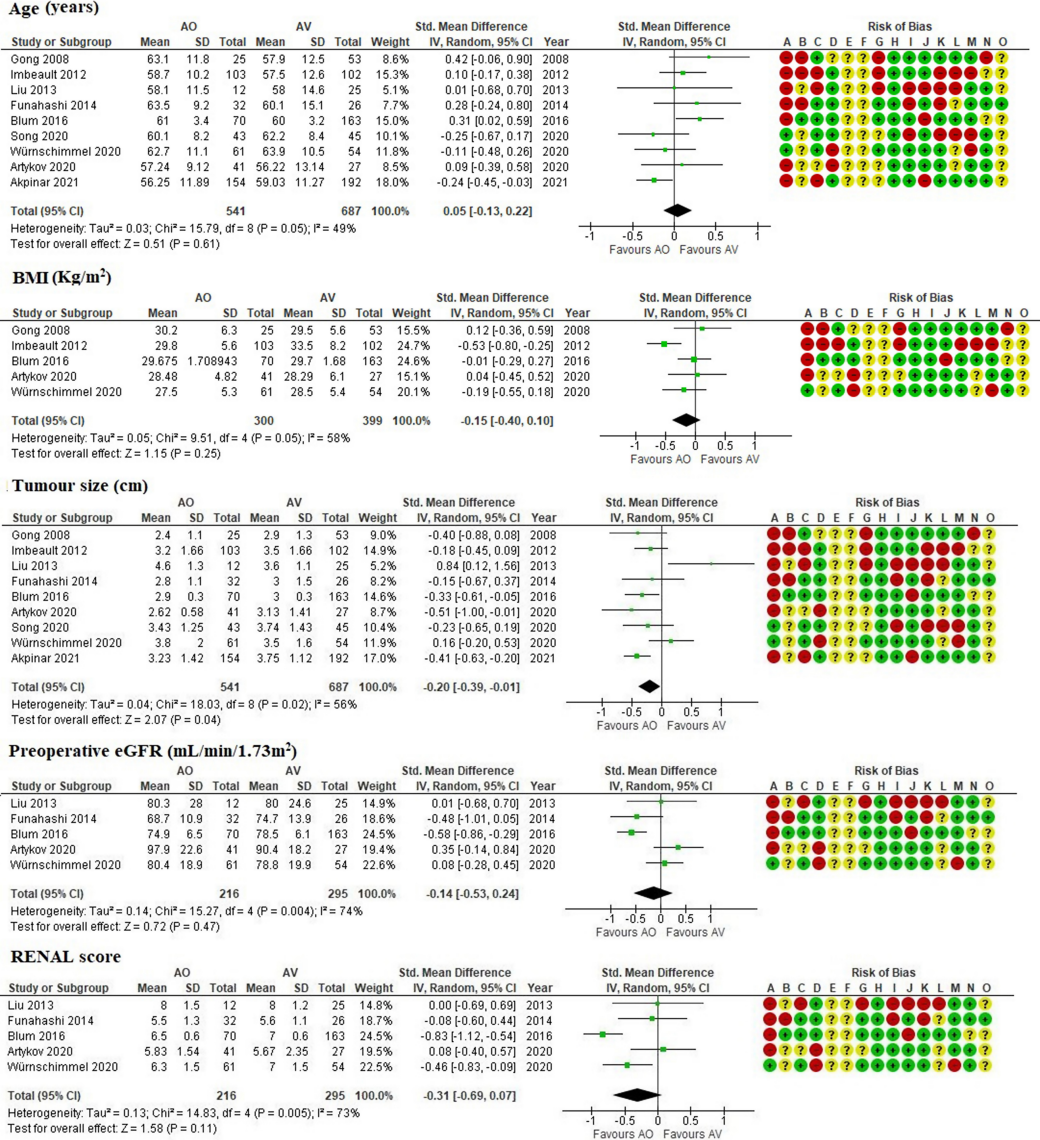
Meta-analysis of patients’ age, body mass index, tumor size, preoperative eGFR, and RENAL score. (A) random sequence generation/balanced groups; (B) allocation concealed/unrelated to confounders; (C) comparable groups at baseline; (D) equal care except for intervention; (E) Blinding participants; (F) blinding carers; (G) equal follow-up; (H) treatment completion; (I) available data of postoperative complications; (J) available data of transfusion rate; (K) available data of CKD; (L) appropriate length of follow-up; (M) precise definition of outcome; (N) valid reliable outcome assessment; (O) blinding of investigators. CKD, chronic kidney disease; eGFR, estimated glomerular filtration rate.

**Patients’ Characteristics:** Figure 4 summarizes the meta-analysis results for age, BMI, tumor size, preoperative eGFR, and renal score. The analysis of overall studies revealed a significant difference between the two groups regarding the tumor size (*P* < .05); however, the effect size was small.

**Figure 5.**
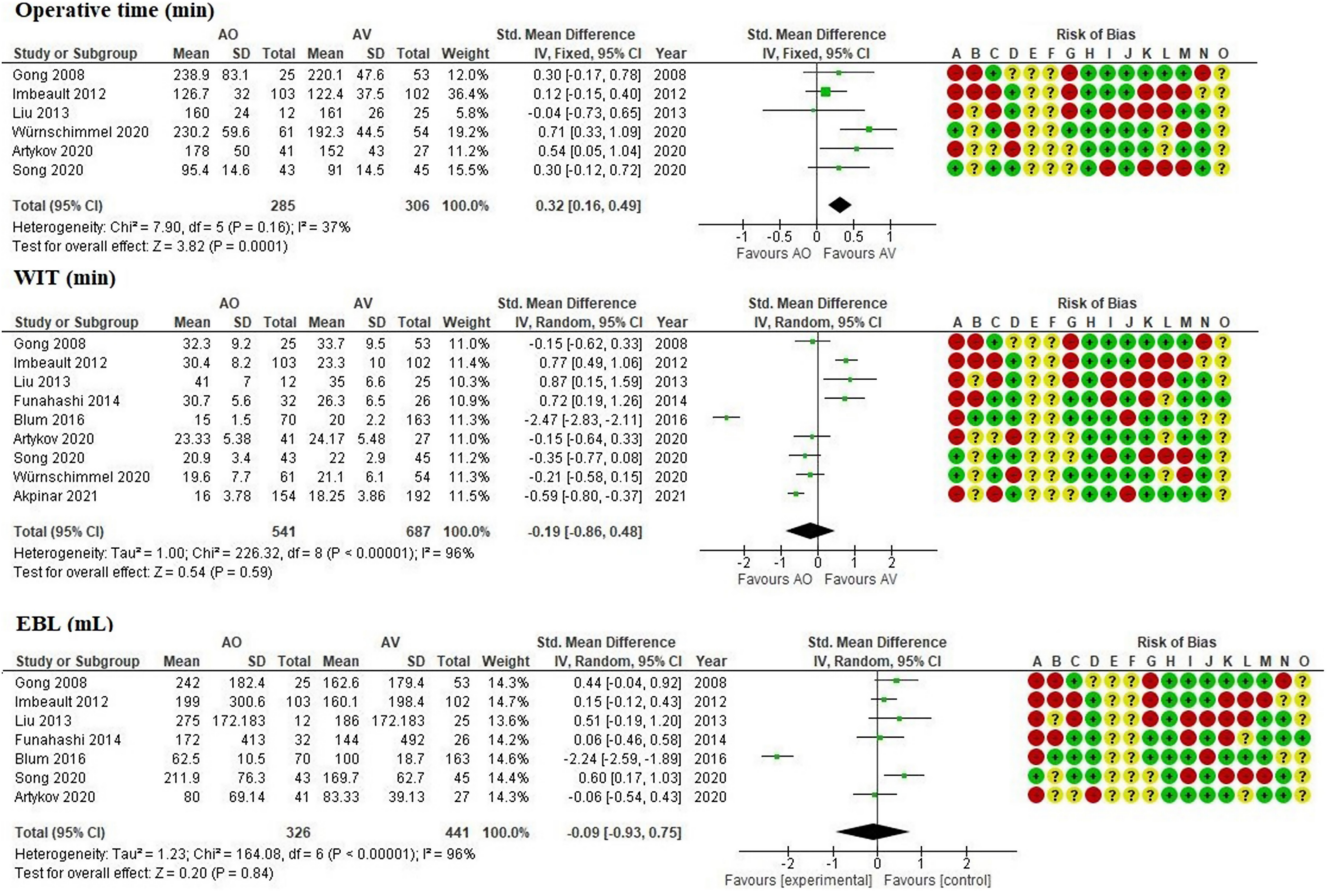
Meta-analysis of operative time, estimated blood loss, and warm ischemia time. (A) random sequence generation/balanced groups; (B) allocation concealed/unrelated to confounders; (C) comparable groups at baseline; (D) equal care except for intervention; (E) blinding participants; (F) blinding carers; (G) equal follow-up; (H) treatment completion; (I) available data of postoperative complications; (J) available data of transfusion rate; (K) available data of CKD; (L) Appropriate length of follow-up; (M) precise definition of outcome; (N) valid reliable outcome assessment; (O) blinding of investigators. CKD, chronic kidney disease.

**Operative Time:** The operative time was reported by 6 studies (see Figure 5). The operative time of the AO clamping group was prolonged in 5 studies,^10,16-19^ but 2 studies only^16,19^ showed a significant effect. The study by Liu et al^20^ showed a slight, nonsignificant reduction of time in the AO group. The pooled SMD of the 6 studies was 0.32 [95% CI: 0.16, 0.49, *P* < .001], indicating a small effect size.

**Warm Ischemia Time:** The warm ischemia time was reported by all the included studies (Figure 5). The warm ischemia time was significantly longer in 3 studies,^4,10,20^ while it was significantly shorter in 1 study^9^ and nonsignificantly shorter in the remaining studies.^15-19^ The pooled SMD of warm ischemia time was −0.19 [95% CI: −0.86, 0.48, *P* = .59] (negligible effect size).

**Estimated Blood Loss:** The amount of estimated blood loss was reported by 7 studies (Figure 5). Four studies showed an increase, though nonsignificant, of blood loss in the AO group.^4,10,17,20^ Song and Jang^18^ showed higher blood loss in the AO clamping group with a significant difference. Two studies^9,16^ showed less blood loss in the AO group, with a significant difference in the study by Blum et al.^9^ The pooled SMD was −0.09 [95% CI: −0.93, 0.75, *P* = .84] (negligible effect size).

**Figure 6.**
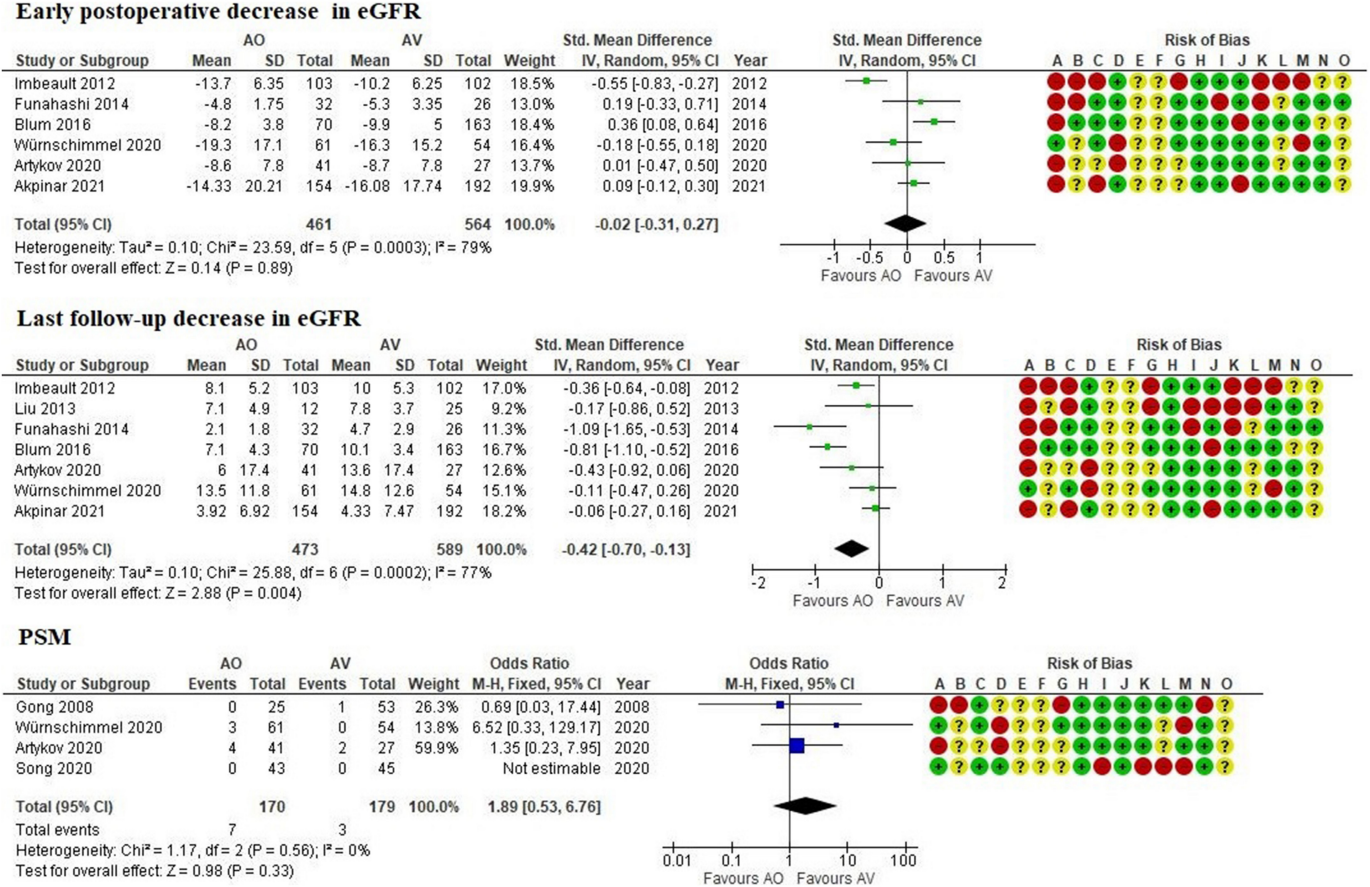
Meta-analysis of postoperative change in eGFR and positive surgical margin. (A) random sequence generation/balanced groups; (B) allocation concealed/unrelated to confounders; (C) comparable groups at baseline; (D) Equal care except for intervention; (E) blinding participants; (F) blinding carers; (G) equal follow-up; (H) treatment completion; (I) available data of postoperative complications; (J) available data of transfusion rate; (K) available data of CKD; (L) Appropriate length of follow-up; (M) precise definition of outcome; (N) valid reliable outcome assessment; (O) blinding of investigators. CKD, chronic kidney disease; eGFR, estimated glomerular filtration rate.

**Positive Surgical Margin:** The incidence of PSM was nonsignificantly higher in the AO clamping group in 2 studies,^16,19^ but lower in the study by Gong et al.^17^ The study by Song and Jang^18^ reported the absence of PSMs in either group (see Figure 6). The pooled OR was 1.89 [95% CI: 0.53, 6.76, *P* = .33] (small effect size).

**Early Postoperative Change in eGFR:** Analysis of the results of 6 studies (Figure 6) showed that the reduction in eGFR during the early postoperative period was higher in the AO clamping group in 2 studies,^10,19^ with 1 study demonstrating a significant difference.^10^ The reduction was lower in the AO group in the remaining 3 studies,^4,9,15,16^ with a significant effect in the study by Blum et al.^9^ The pooled SMD was −0.02 [95% CI: −0.31, 0.27, *P* = .89] (negligible effect size).

**Change in eGFR on the Last Follow-Up:** The analysis of the reduction in eGFR on the last follow-up visit is demonstrated in Figure 6. The reduction was lower in the AO group in 7 studies: 3 showing a significant difference^4,9,10^ and 4 studies with a nonsignificant effect.^15,16,19,20^ The pooled SMD was −0.42 [95% CI: −0.70, −0.13, *P* = .004] (small effect size).

**Figure 7.**
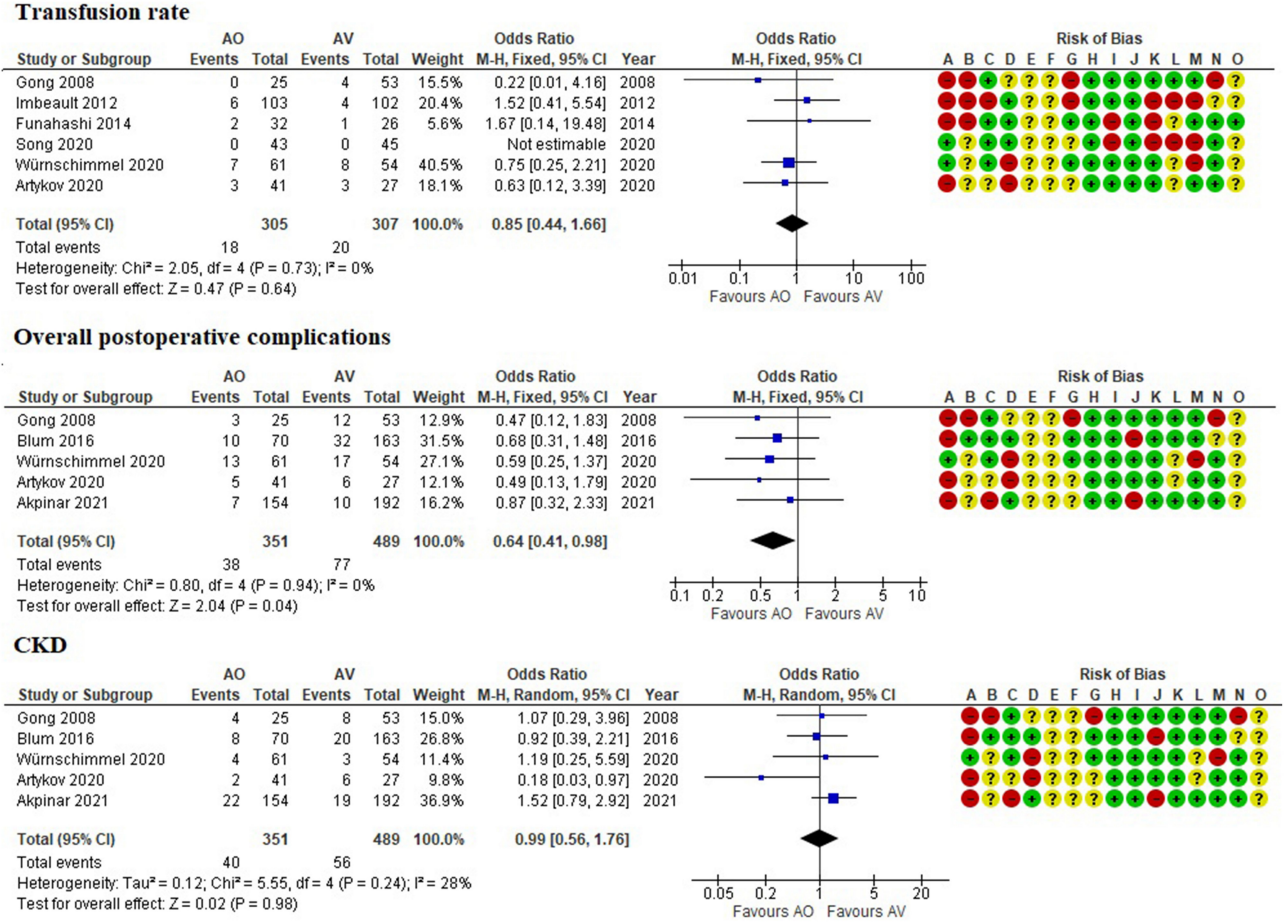
Meta-analysis of transfusion rate, overall postoperative complications, and progression to CKD. (A) Random sequence generation/balanced groups; (B) allocation concealed/unrelated to confounders; (C) comparable groups at baseline; (D) equal care except for intervention; (E) blinding participants; (F) blinding carers; (G) equal follow-up; (H) treatment completion; (I) available data of postoperative complications; (J) available data of transfusion rate; (K) available data of CKD; (L) appropriate length of follow-up; (M) precise definition of outcome; (N) valid reliable outcome assessment; (O) blinding of investigators. CKD, chronic kidney disease; eGFR, estimated glomerular filtration rate.

**Transfusion Rate:** The rate of blood transfusion was nonsignificantly higher in the AO group in 2 studies,^4,10^ but nonsignificantly lower in 3 studies.^16,17,19^ Song and Jang^18^ reported that none of their patients required blood transfusion. The pooled OR was 0.85 [95% CI: 0.44, 1.66, *P* = .64] (negligible effect; Figure 7).

**The Overall Rate of Postoperative Complications:** The rate of postoperative complications was reported to be nonsignificantly lower in the AO clamping group in 5 studies.^9,15-17,19^ The pooled OR was 0.64 [95% CI: 0.41, 0.98, *P* = .04] (negligible effect size; Figure 7).

**Progression to CKD:** Three studies showed a nonsignificantly higher rate of CKD development in the AO clamping group,,^15,17,19^ while Blum et al^9^ showed a nonsignificant decrease in this group and only the study by Artykov et al^16^ showed a significant reduction of the rate of CKD in the AO group. The pooled OR for the 5 studies was 0.99 [95% CI: 0.56, 1.76, *P* = .98] (indicating a negligible effect size; Figure 7).

## Discussion

### Summary of the Main Findings

The use of clamping techniques during PN has been a concern regarding its effect on the residual renal tissue and renal function after surgery.^21^ Although the zero-clamping technique has been used in PN to avoid this risk, the results show that it is not superior to on-clamping techniques in preserving renal functions.^22,23^ Moreover, clamping allows for better control and visualization during resection of renal tumors, which in turn affects the amount of residual renal parenchyma and consequently impacts the functional outcome following surgery. Controversial results were published regarding the most suitable clamping technique in terms of preserving renal function. Therefore, this meta-analysis was conducted to synthesize the published evidence regarding the use of AV clamping or AO clamping during PN.

The conducted literature search yielded 9 studies that conformed with the eligibility criteria of this meta-analysis.^4,9,10,15-20^

As regards the effect on renal function, all included studies assessed different parameters. Serum creatinine and creatinine clearance only were used for renal function assessment by Gong et al^17^; however, these 2 measurements are not considered accurate for assessing renal function as they do not consider the patient’s age, sex, and race—factors that are incorporated in the estimation of eGFR. Seven studies depended mainly on the reduction in eGFR after surgery, which is considered a more accurate marker for renal function than creatinine. The present meta-analysis found that the early reduction percentage in eGFR during the early postoperative period was nonsignificantly lower in the AO group. However, the reduction in eGFR at the last follow-up was significantly lower in the AO clamping group (SMD: −0.42 [95% CI: −0.70, −0.13, *P* = .004]. This suggests that AO clamping preserves renal parenchyma during PN and results in a better long-term functional outcome. Previous studies hypothesized that such a beneficial effect may be due to the retrograde venous blood flow that provides partial oxygenation of the renal tissues during the time of AO clamping.^24,25^

However, the analysis of the change in eGFR was based on 7 studies only that had widely varying ranges of follow-up (from approximately 2 months up to 65 months after surgery). Besides, the duration of follow-up was much shorter for the AV clamping group in some of these studies,^10,20^ which may provide less time for recovery of eGFR than that observed in the AO clamping group.

Meanwhile, the use of eGFR does not consider the compensatory effect exerted by the nonoperated kidney. Therefore, it is recommended to perform a split renal function assessment. Unfortunately, only 4 studies^4,10,18,19^ used renal scintigraphy to assess the change in renal function after surgery. The discrepancies in the used methods and units for renal function across the studies made the pooling of their results improper, thus no meta-analysis was conducted on this important parameter. Nevertheless, the results of the 4 studies showed the lack of significant difference between the 2 methods of clamping. Likewise, the rates of development or progression of CKD after surgery were similar in both groups, with OR around one and no significant difference.

Several factors besides the clamping technique interact with each other in patients undergoing PN and can impact renal function after surgery. The warm ischemia time was identified among the strongest risk factors that greatly influence renal function.^10,26^ The differences in warm ischemia time may explain the controversial results of functional outcomes following PN.^27^ A warm ischemia time longer than 30 minutes during LPN has been associated with a higher reduction in postoperative split renal function.^28^ Blum et al^9^ and Akpinar et al^15^ attributed the absence of protective effect in the AO clamping group in their cohorts to the relatively short warm ischemia type in both clamping techniques. It was suggested the RAPN is associated with a shorter warm ischemia time^28^; thus, studies that included a subset of patients with RAPN may show a better functional outcome regardless of the clamping technique. On the other hand, warm ischemia time seems to be prolonged with AO clamping, particularly during LPN, presumably due to a higher EBL and less clear visualization of tumor margins. However, the present meta-analysis did not find a significant difference in EBL or warm ischemia time between the two groups.

Another factor that potentially affects the functional outcomes following PN is the surgical approach. Most studies that demonstrated a beneficial effect of AO clamping on renal function were animal models with open PN models.^6,7^ However, studies on humans in which RAPN and – to a lesser degree—LPN were used tended to show no significant difference in renal function.^9,10,15,19^ This variation in outcomes across the different surgical approaches was attributed to the compression on renal veins caused by pneumoperitoneum, which negates retrograde venous blood flow during LPN and RAPN.^4,24^

The present study also assessed the safety of the 2 clamping techniques. The pooled overall rate of complications was significantly lower in the AO clamping group (OR = 0.64 [95% CI: 0.41, 0.98], *P* = .04).

It was commonly thought that AO clamping may add to the operative difficulties through venous backflow bleeding that reduces the quality of visualization. However, the results of this meta-analysis revealed that though the operative time tended to be longer with AO clamping, the effect size was small. Four out of 6 studies^10,16-18^ reported a tendency of a nonsignificantly longer operative time with AO. One study only^19^ stated that time was significantly longer with AO clamping, while only Liu et al^20^ reported a comparable operative time in the 2 groups. The significant difference found by Würnschimmel et al^19^ may be attributed to the different surgical approaches between the 2 groups as one group underwent LPN while the other RAPN. The small effect of AO clamping on operative time is also supported by the lack of significant differences between the 2 groups regarding the warm ischemia time, EBL, and transfusion rate, indicating that the magnitude of backward venous pressure during AO clamping would not result in a severer hemorrhage that may obscure the operative field.

The difficulties added by AO clamping were suggested before to contribute to a higher rate of PSM in the case of PN for malignant renal tumors. A higher rate of PSM has been associated with increased risk of recurrence and distant metastasis as well as lower survival rates,^29^ thus it is of utmost importance to improve conditions that maximize the complete resection of malignant tumors to achieve negative surgical margins. Only 4 studies out of the included 9 in this meta-analysis commented on the rate of PSM and no significant difference was observed despite a slight tendency to a higher rate with AO clamping. To decide whether this difference is clinically significant or not, the conduction of long-term studies is warranted to record the oncological outcomes including the rate of recurrence, distant metastases, and cancer-specific survival rates.

### Overall Completeness, Applicability, and Quality of the Evidence

This meta-analysis summarized the current evidence on the efficacy and safety of AO clamping during PN compared to AV clamping. The 2 clamping techniques were comparable regarding their safety, but the AO clamping technique may have a renoprotective effect. However, deriving a clinical recommendation based on this observed protective effect is subject to some cautious considerations due to the limitations of the included studies.

The studies showed some variations in their inclusion criteria as well as the used surgical technique for PN. Some studies included malignant tumors only and specified the stage of cancer.^16,18,19^ Other studies included only patients who underwent open,^18,20^ minimally invasive PN,^4,9,10,17^ or a sample of both techniques.^15^ Funahashi et al^4^ excluded endophytic and hilar renal tumors. In addition to these variations, other variable patients’ and surgeons’ characteristics were adopted for inclusion/exclusion in these studies, which are expected to cause heterogeneity across the included studies.

Heterogeneity across the included studies was observed in baseline characteristics (age, tumor size, BMI, preoperative eGFR, and renal score). A larger BMI and tumor size in the AV clamping group could add to the difficulties encountered during surgery and to the amount of resected renal parenchyma, thus affecting renal function.^30^ The differences in baseline characteristics may be due to the preference of the surgical team (in the cohort studies) to use AV clamping with cases expected to show some technical challenges as clamping of both artery and vein has been associated with less EBL and thus allows better visualization of the surgical field.

In addition, 5 of the included studies were retrospective in design,^9,10,15-17^ resulting in a high risk of blinding of patients and assessors as well as the disparities in the duration of follow-up both across and within the studies. All these factors could potentially affect the results of this meta-analysis. In addition, the most appropriate method of assessing renal function (renal scintigraphy to assess split function) was used in a few studies only that reported the lack of significant difference.

### Agreements and Disagreements with Other Studies or Reviews

A previous meta-analysis by Cao et al^27^ addressed the same research question, but they included only 5 studies,^4,9,10,17,20^ while the remaining 4 studies assessed in the present meta-analysis were published later. Their results were similar to the present study, showing the lack of significant differences as regards the warm ischemia time, EBL, and transfusion rate. The results of assessing renal function based on the change in eGFR were also similar. However, we found that the operative time was significantly longer in the AO clamping group. The present meta-analysis endeavored to fill a gap of knowledge as the previous meta-analysis by Cao et al^27^ did not discuss some clinically relevant outcomes as the incidence of PSM or newly developed or progression of CKD, which represent important concerns in these cases.

### Limitations

The present study has potential limitations. Firstly, articles published only in English were included. Secondly, conference abstracts were excluded. These 2 factors may have deprived the analysis of studies providing variable data which may alter the results. Also, the authors of the included studies were not contacted to inquire about unpublished data. It is recommended that a meta-analysis addressing this research question in the future should attempt to obtain the data from the authors of non-English published articles and conference abstracts. Moreover, the relatively low number of retrieved studies prevented the conduction of subgroup analyses based on the surgical approach or type of studies.

## Conclusions, Implications for Practice, Policy, and Future Research

The results of this meta-analysis show that AO clamping is a safe technique that would not add to the difficulties of PN or increase its rate of complications compared to AV clamping. Moreover, AO clamping may be preferable to the AV clamping technique as it may produce a protective effect on renal function, particularly on the long-term functional outcomes. However, due to limitations of the included studies, the conduction of large size randomized clinical trials with a long duration of follow-up and with blinded assessment of the functional and oncological outcomes is required before recommending the replacement of AV clamping with AO clamping during PN.

## Figures and Tables

**Table 1.** The Settings and Eligibility Criteria of the Included studies (n = 9)

**Studies**	**Design and settings**	**Surgical Approach**	**Inclusion Criteria**	**Exclusion Criteria**
Gong (2008)	- Retrospective cohort - Single center in the United States - From October 2002 to May 2006	LPN	Patients undergoing LPN	Previous or subsequent extirpative renal surgeries Patients with open conversion were excluded from the analysis of PO renal function.
Imbeault (2012)	- Retrospective cohort - Single center in Canada - From March 2003 to December 2008	Transperitoneal LPN	Surgically eligible patients with localized enhancing renal masses undergoing LPN	No absolute exclusions
Liu (2013)	- Prospective cohort - Single center in the United States - From March 2009 to July 2011	Open PN	Patients with potentially malignant renal mass on CT/MRI undergoing complex open PN and were unfit for LPN or RAPN due to tumor size or location	None
Funahashi (2014)	- Prospective cohort - Single center in Japan - From August 2005 to January 2013	Transperitoneal LPN	Patients with nonhilar exophytic renal tumors undergoing LPN	Endophytic and hilar tumors
Blum (2016)	- Retrospective cohort - Multicenter in the United States - From 2008 to 2016	Transperitoneal RAPN	Patients with a solitary T1 renal mass undergoing PN and had a functional contralateral kidney, baseline eGFR ≥ 30, and follow-up data available between 3 and 18 months post-RAPN	Surgeons performing AV clamping in <10% of cases
Artykov (2020)	- Retrospective cohort - Single center in Turkey - From 2008 to 2019	Transperitoneal LPN/RAPN	Patients with solitary, unilateral, cT1 renal masses undergoing LPN/RAPN	Off-clamp partial nephrectomies, conversion to open surgery, compelled RN, missing clamping data & lost to follow-up
Song (2020)	- Randomized controlled trial - Single center in Korea - From 2015 to 2018	retroperitoneal open PN	Patients with T1 renal tumor undergoing open PN	None
Würnschimmel et al^19^	- Randomized clinical trial - Single center in Switzerland - From 2015 to 2019	LPN with AV clamping or RAPN with AO clamping	Patients with cT1-T2 renal masses	CCI >10, CKD stages 4-5, previous renal surgeries or concomitant oncological diseases, immune diseases, cT3+ or cN1 renal cancer
Akpinar (2021)	- Retrospective cohort - Single center in Turkey - From January 2011 to January 2018	Transperitoneal LPN/RAPN or open PN	Patients who underwent open or minimally invasive PN	Anatomical malformation of kidney, preoperative CKD, conversion to RN, undergoing simultaneous intraabdominal surgeries, bilateral renal masses, solitary kidney, follow-up <2 years, and zero or segmental ischemia

AO, renal artery only clamping; AV, renal artery and vein clamping; CCI, Charlson Comorbidity Index; CKD, chronic kidney disease; CT, computed tomography; eGFR, estimated glomerular filtration rate; LPN, laparoscopic partial nephrectomy; MRI, magnetic resonance imaging; PO, postoperative.

**Table 2.** The sample size, assessment of renal outcome, and follow-up of patients in the included studies (n = 9)

**Studies**	**AO (Number)**	**AV (Number)**	**Assessment of Renal Outcome**	**Follow-Up AO/AV (Months)**
Gong (2008)	25 patients	53 patients	- Cr and CrCl (preoperatively, immediately after surgery, on POD1, and at the time of last follow-up). - CKD was defined as either Cr>1.4 mg/dl or CrCl<60 ml/min.	21.9 ± 11.8/10.1 ± 9.9 (mean ± SD)
Imbeault (2012)	103 patients	102 patients	- Cr changes and eGFR (preoperatively and at POD1, 3 months, and at the last follow-up) - Split renal function using renal MAG-Lasix scintigraphy (pre- & postoperative). - CKD not assessed	44 (29-65)/15 (2-28) Median (min-max)
Liu (2013)	12 patients	25 patients	- Renal function: Cr and eGFR (preoperative, postoperative, and last follow-up)	6.8/2.7 (median)
Funahashi (2014)	32 patients	26 patients	- Serum Cr and eGFR (preoperatively, 1 week, and 6 months postoperatively) - 99mTc-MAG3 scintigraphy (preoperatively, 1 week, and 6 months postoperatively) - CKD not assessed	6 months
Blum (2016)	70 patients	163 patients	- Percent change in eGFR, and AKI at discharge - Percent change in eGFR and progression to CKD at 9 months - Progression to CKD was defined as an increase from CKD stage 1 or 2 to CKD stage ≥3 or an increase from CKD stage 3 to CKD stage ≥4 at a median follow-up of 9 months.	9.3/8.7 (median)
Artykov (2020)	41 patients	27 patients	- Cr and eGFR - CKD was defined as eGFR<60 ml/min/1.73 m^2^	13.5 (9–44.5) Median (IQR)
Song (2020)	43 patients	45 patients	- Cr: 1 month before surgery then POD 1, 7, 15, and 1st and 3rd months - Differential renal function: 3 months after surgery. - CKD was not assessed	3 months
Würnschimmel (2020)	61 patients	54 patients	- eGFR and MAG3 renal scintigraphy preoperatively and at 6 months follow-up. - CKD was not defined	6 months
Akpinar (2021)	154 patients	192 patients	- eGFR: at the 6th, 12th, and 24th months - the percentage change of renal function compared with baseline eGFR - Progression to CKD was defined as an increase from baseline CKD stage 1 or 2 to ≥3.	24 months

AO, renal artery only clamping; AV, renal artery and vein clamping; CKD, chronic kidney disease; Cr, creatinine; CrCl, creatinine clearance; eGFR, estimated glomerular filtration rate; IQR, interquartile range; MAG, mercaptoacetyl triglycine; PN, partial nephrectomy; POD, postoperative day; SD, standard deviation.
